# Relationships Among Extreme Sports Participation, Sensation Seeking, and Negative Risky Behaviors of Middle-School Students

**DOI:** 10.3389/fpsyg.2021.722769

**Published:** 2021-08-30

**Authors:** Hu Chunmei, He Lingling, Ge Ning, Li Yang

**Affiliations:** ^1^Laboratory of Emotion and Mental Health, Chongqing University of Arts and Sciences, Chongqing, China; ^2^Aviation and Automobile School, Chongqing Youth Vocational and Technical College, Chongqing, China

**Keywords:** extreme sports participation rate, sensation seeking, smoking, gambling, middle-school students, drinking alcohol

## Abstract

**Objective:** The aim was to investigate the relationships among extreme sports participation, sensation seeking, and negative risky behaviors (smoking, drinking alcohol, and gambling) for middle-school students.

**Methods:** Using a convenience sampling procedure, all students from a middle school in a district of Chongqing were selected to participate in the survey, which included questions on their extreme sports participation rate, and smoking, drinking alcohol, and gambling behavior.

**Results:** A sample of 2,987 middle-school students participated in this study. The results showed that the proportions of students participating in extreme sports, smoking, drinking alcohol, and gambling were 19.9, 4.8, 18.4, and 3.0%, respectively. There were significant differences between different genders, schools, place of residence, smoking, drinking, gambling, and sensation seeking of the participation rate of students of extreme sports, the rate of boys, junior middle-school students, urban students, smokers, alcohol drinkers, gamblers, and high-sensation-seeking students were relatively higher than that of girls, senior middle-school students, rural students, no-smokers, no-alcohol drinkers, no-gamblers, and low-sensation-seeking students. Alcohol drinking, gambling, and sensation seeking were associated with extreme sports participation, and the students who drank alcohol, who gambled, and who were high sensation seeking were more likely to participate in extreme sports than those who did not drink alcohol, who did not gamble, and who were low sensation seeking.

**Conclusion:** Middle schools should integrate extreme sports education into physical education and risky-behavior education, strengthen relevant knowledge and safety training, and guide students to meet their sensation-seeking needs through participation in extreme sports instead of risky behaviors.

## Introduction

### The Development of Extreme Sports in China

Extreme sports took off in the United States in the 1960s and were introduced in China in the 1990s. Since then, extreme sports have developed rapidly in China. Especially after bicycle motocross (BMX), racing was included in the Beijing Summer Olympics in 2008, a growing number of people are familiar with and love extreme sports. The number of participants keeps increasing, and the number of commercial extreme sports venues and clubs has increased rapidly (Zhao and Wang, 2009).

Extreme sports emphasize participation, entertainment, and the spirit of courage, which fully meet the characteristics of young people who want to show themselves, highlight their personalities, be free from restrictions, and like fashionable things. Thus, the main participants in extreme sports are young people (Xue and Yan, 2018). In many universities and primary and middle schools in China, some kinds of extreme sports have been widely developed, such as indoor rock climbing, skateboarding, parkour, and roller skating (Zhao and Wang, 2009). Compared with wingsuit flying and outdoor rock climbing, extreme sports that are popular in schools have few requirements in terms of the venues and equipment needed to participate. They are also low risk and relatively simple to learn, so they attract more adolescents to join in.

### Extreme Sports and Sensation Seeking

Sensation seeking is a personality trait defined as searching for experiences and feelings that are “varied, novel, complex, and intense.” It is also understood as the readiness to “take physical, social, legal, and financial risks for the sake of such experiences” (Zuckerman et al., 1993). The development of sensation seeking is closely related to adolescent pubertal maturation. Sensation seeking develops into an inverted U-shaped curve, increasing in early adolescence from age 10 to 13, peaking in the middle adolescence at age 14 or 15, and decreasing steadily thereafter (Steinberg et al., 2008). These increases of sensation seeking are thought to be linked to an increase and then decline in the prefrontal and paralimbic dopaminergic activity during the period following puberty. Sensation seeking among Chinese adolescents also increases from early adolescence, peaks in middle adolescence, and then decreases gradually. During this decline, however, there is an obvious rebound from ages 17 to 20, but the slight increase is still lower than the original peak (Hu et al., 2018).

In China, there are three major systems of basic and secondary education: primary school (grades 1–6), junior middle school (grades 7–9), and senior middle school (grades 10–12). Children must be above 6 to attend primary school. So, we can see that the middle-school students in China are in early adolescence (from age 10 to 13) and middle adolescence (from age 14 to 17) (Shulamn et al., 2016). In this stage, with the increase of sex hormone secretion, prefrontal and paralimbic dopaminergic activity increases, and the level of sensation seeking will increase until it reaches its peak. This leads the students to engage in stimulating, adventurous, and fashionable behaviors to satisfy their sensation-seeking needs (Collado et al., 2014). Sensation seeking positively predicts smoking, alcohol use, marijuana use, and unsafe sex among high-school students, with students exhibiting higher levels of sensation seeking, practicing more of these behaviors (Cohen and Fromme, 2002; Ye et al., 2011; Kong et al., 2013). Sensation seeking can be used as an indicator for screening risky behaviors (Safa et al., 2019).

Engaging in highly stimulating, potentially dangerous physical activity is one of how socially acceptable sensation seeking can be acquired (Malkin and Rabinowitz, 1998), and extreme sports are perfect examples of this. Researchers have conducted studies on the relationship between extreme sports and sensation seeking and have found that sensation seeking can predict the performance in higher-risk discipline freediving but not the performance in lower-risk disciplines (Baretta et al., 2017). Cliff and free divers are sensation seekers, and there are no differences with respect to sensation-seeking behavior that can be found between men and women participants (Frick, 2021). The sensation-seeking of college students is the strongest positive predictor of extreme sports participation (Weishaar et al., 2021). So far, no study has explored the relationship between the participation of middle-school students in extreme sports and sensation seeking. These individuals are experiencing the highest level of sensation seeking in their lives. With the rapid development of extreme sports in China, more students are getting in touch with these needs and participating in extreme sports. Does the sensation-seeking of middle-school students also affect their participation in extreme sports? This is the question that is explored in this study.

### Extreme Sports and Risk Behaviors

Risk behaviors refer to purposive participation of an individual in some activity that involves potential negative consequences or losses (social, monetary, interpersonal), as well as involves perceived positive consequences or gains (Ben-Zur and Zeidner, 2009), and they can be categorized as negative or positive risk behaviors (Ozmen and Sümer, 2011). Negative risky behaviors refer to those that are not accepted by society and have a negative impact on physical and mental health, such as smoking, drinking alcohol, and unsafe sex, among others. Positive risky behaviors are those that are widely accepted by society and can promote physical and mental health by taking certain protective measures and training, such as extreme sports. Researchers have carried out a large number of studies on the negative risky behaviors of adolescents (e.g., smoking, drinking alcohol, gambling, consuming energy drinks, etc.) and have found that the negative risky behaviors tend to cluster; in other words, when one of these behaviors is displayed, it is likely that others are involved as well (Yang et al., 2016; Hu and Qi, 2020). Relatively few studies have been conducted on the positive risk behaviors, and their positive role in adolescent development has been largely ignored (Tian et al., 2016).

Extreme sports are considered socially acceptable positive risk behaviors. If certain protective measures and training are taken in the process of participating in positive risk behaviors, the healthy development of the bodies and minds of adolescents can be promoted (Tian et al., 2016). A large proportion of extreme-sport enthusiasts, especially younger adults, regularly consume energy drinks (Goodhew et al., 2020). We know that drinking energy drinks, smoking, drinking alcohol, gambling, and other negative risk behaviors will cluster. Thus, we should further explore whether the participation of middle-school students in extreme sports also clusters with these negative risk behaviors.

At present, there are a few studies on extreme sports participation among middle-school students. This study investigates the participation rate in extreme sports among middle-school students and investigates the relationships among extreme sports participation, sensation seeking, and negative risk behaviors. The results have value for schools in regard to carrying out extreme sports education. Smoking, drinking alcohol, and gambling are the common negative risk behaviors among Chinese middle-school students (Hu et al., 2017). Therefore, this study investigates the relationships among these three negative risk behaviors and the participation of students in extreme sports.

Based on the existing relevant studies, we make the following research hypotheses. Hypothesis 1: sensation seeking was associated with extreme sports participation; students with a high sensation seeking were more likely to participate in extreme sports. Hypothesis 2: smoking, drinking alcohol, and gambling were associated with extreme sports participation; these negative risk behaviors cluster with the participation in extreme sports.

## Materials and Methods

### Participants

A total of 3,244 questionnaires were sent out to all students at a middle school in a district of Chongqing using convenience sampling from March to April 2021. All students who participated in the survey signed an informed consent form before completing the questionnaire.

Before the survey was administered, psychological investigators contacted the middle school, and the school organized the teachers to participate in an informational meeting. During the meeting, the investigator described the content and purpose of the survey to the teachers. After the meeting, the investigators engaged in discussion with the teachers to determine the specific time to send questionnaires to each class. During the survey, the teachers led the investigators to each classroom. The investigator first informed the students about the content and purpose of the investigation, and then, the students were asked to read the informed consent form and sign it voluntarily. After doing so, the investigators distributed the questionnaires, read instructions aloud, and reminded the students to answer truthfully. It took about 10 min for the students to complete the questionnaire. After completing the questionnaire, the investigators immediately withdrew the documents. Then, the investigators sorted the questionnaires, eliminated the invalid questionnaires, and carried out the data entry and analysis.

### Measures

In addition to sociodemographic characteristics, including gender, school type (junior/senior middle school), and place of residence (urban/rural), three instruments were used in this study.

#### Extreme Sports Participation Rate Scale

The extreme sports participation rate was measured by asking a question: “Have you participated in extreme sports in the past 12 months, such as indoor rock climbing, skateboarding, parkour, and roller skating, and so on?” As there was limited variability in the rate of extreme sports participation, the answer was dichotomized to indicate participation (once or more) or no participation in extreme sports in the past 12 months. Answering “yes” means the student had participated in extreme sports once or more times while answering “no” means the student had never participated in extreme sports in the past 12 months. The item was scored as either Yes (coded 1) or No (coded 0).

#### Sensation-Seeking Scale

The scale is a subset of 11 items from the Impulsive Sensation-Seeking Scale (ImpSS). ImpSS was developed by Zuckerman et al. (1993). It consists of 19 items and two subscales; 11 items measure sensation seeking and eight items measure impulsivity. The two subscales have shown good internal consistency (alphas ranging from 0.64 to 0.77; Zuckerman, 2002; Kong et al., 2013). The Chinese version of the sensation-seeking scale was revised by Hu Chunmei, and it has good validity in the surveys of Chinese adolescents (alphas ranging from 0.71 to 0.75; Hu et al., 2017; Hu and Wang, 2020). The items were scored as either Yes (coded 1) or No (coded 0). The higher the total score of the scale, the higher the level of individual sensation seeking. We used sensation seeking as a continuous variable in the logistic regression. To compare differences in the extreme sports participation rate with different sensation-seeking levels, we divided the participants into two groups (high and low sensation seeking). High sensation seeking was defined as the sensation-seeking scores above 5.0, which corresponded to the 75th percentile in this study (Lee et al., 2016). In this survey, the scale was valid and reliable, with Cronbach's alpha of 0.77, and scores ≤ 5.0 were indicative of low sensation seeking.

#### Negative Risk Behavior Scale

Three items measured the incidence of three negative risk behaviors using yes/no questions, respectively: (1) “Have you actually smoked (not tried) in the past 30 days?”; (2) “Have you actually drunk alcohol (not tried) in the past 30 days?”; and (3) “Have you gambled in the past 30 days? (gambling refers to participating in entertainment in which money, tokens, or other goods are wagered).” The answers were dichotomized to indicate participation (once or more) or no participation in the three negative risk behaviors. Answering “yes” means the student had participated in the risky behavior once or more while answering “no” means they had never participated. The items were scored as either Yes (coded 1) or No (coded 0). The scale was developed by Kong et al. (2013) and was revised by Hu et al. (2017).

### Analysis

Descriptive statistics, including means, SDs, frequencies, and percentages, were calculated on raw data. Chi-squared tests were used to explore the differences in extreme sports participation among middle-school students with different characteristics. Logistic regression analysis was used to study the relationship between sensation seeking, drinking alcohol, gambling, and participation in extreme sports. The data were analyzed using SPSS (version 21).

## Results

### Sample Characteristics

A sample of 3,244 participants completed the questionnaires. The invalid questionnaires (*n* = 257) with missing answers were eliminated, and 2,987 valid questionnaires were collected, with an effective response rate of 92.08%. Around half of the participants were women (*n* = 1,523, 51.0%), junior middle-school students (*n* = 1,521, 50.9%), and rural students (*n* = 1,563, 52.3%). The average age was 15.73 years (SD = 1.90). Table 1 reports further details.

**Table 1:** Sample characteristics (*N* = 2,987).

	**Variable**	**Frequency (%)**
1	**Gender**	
	Male	1,464 (49.0%)
	Female	1,523 (51.0%)
2	**School type**	
	Junior middle school	1,521 (50.9%)
	Senior middle school	1,466 (49.1%)
3	**Place of residence**	
	Rural	1,563 (52.3%)
	Urban	1,424 (47.7%)

### Participation Rate in Extreme Sports, Smoking, Drinking Alcohol, and Gambling

The results showed that 2,392 (80.1%) participants had not participated in extreme sports, and 595 (19.9%) had participated. Furthermore, 2,845 (95.2%) participants had not smoked, while 142 participants (4.8%) had smoked; 2,438 (81.6%) had not drunk alcohol, while 549 (18.4%) had drunk; and 2,896 (97.0%) participants had not gambled, while 91 (3.0%) had gambled.

### Sensation Seeking

The results for sensation-seeking showed that the mean score was 3.59 (SD = 2.70). The number and percentage of participants classified as high sensation seeking were 685 (22.9%); 2,302 (77.1%) were classified as low sensation seeking.

### Differences in Extreme Sports Participation Rate According to the Characteristics of Participants

The results of the chi-square tests indicated that there were significant differences in extreme sports participation rates based on different genders, school types, native places, smoking, alcohol drinking, gambling, and sensation seeking (all *p* < 0.001). The extreme sports participation rates of boys, junior middle-school students, urban students, smokers, alcohol drinkers, gamblers, and high sensation seekers were relatively higher than that of girls, senior middle-school students, rural students, no-smokers, no-alcohol drinkers, no-gamblers, and low-sensation-seeking students. Table 2 details these results.

**Table 2:** The difference in extreme sports participation rate according to the characteristics of participants.

	**Frequency (%)**	**No participation frequency (%)**	**Participation frequency (%)**	*****χ^2^*****
**Gender**				
Male	1,464 (49.0%)	1,132 (77.3%)	332 (22.7%)	13.691^***^
Female	1,523 (21.0%)	1,260 (82.7%)	263 (17.3%)	
**School type**				
Junior middle school	1,521 (50.9%)	1,146 (75.3%)	375 (24.7%)	43.561^***^
Senior middle school	1,466 (49.1%)	1,246 (85.0%)	220 (15.0%)	
**Place of residence**				
Rural	1,563 (52.3%)	1,306 (83.6%)	257 (16.4%)	24.846^***^
Urban	1,424 (47.7%)	1,086 (76.3%)	338 (23.7%)	
**Smoking**				
No smoking	2,845 (95.2%)	2,302 (80.9%)	543 (19.1%)	26.066^***^
Smoking	142 (4.8%)	90 (63.4%)	52 (36.6%)	
**Alcohol drinking**				
No drinking	2,438 (81.6%)	2,020 (82.9%)	418 (17.1%)	64.009^***^
Drinking	549 (18.4%)	372 (67.8%)	177 (32.2%)	
**Gambling**				
No gambling	2,896 (97.0%)	2,340 (80.8%)	556 (19.2%)	30.957^***^
Gambling	91 (3.0%)	52 (57.1%)	39 (42.9%)	
**Sensation seeking**				
Low	2,302 (77.1%)	1,939 (84.2%)	363 (15.8%)	108.417^***^
High	685 (22.9%)	453 (66.1%)	232 (33.9%)	

*** *p < 0.001*.

### Relationships Between Sensation Seeking, Smoking, Drinking Alcohol, Gambling, and Extreme Sports Participation

The relationships between sensation seeking, smoking, drinking alcohol, gambling, and extreme sports participation were determined using the logistic regression analysis. In the regression, gender, school type, place of birth, smoking, alcohol drinking, gambling, and sensation seeking were used as independent variables, and extreme sports participation was the dependent variable. The results indicated that the extreme sports participation rate was associated with drinking alcohol, gambling, and sensation seeking after controlling for gender, school type, and place of residence. The students who drank alcohol, gambled, and were high sensation seeking were more likely to participate in extreme sports than those who did not drink alcohol, gamble, and were low sensation seeking. The results are presented in Table 3.

**Table 3:** Logistic regressions associating with extreme sports participation rate, sensation seeking, smoking, drinking alcohol, and gambling.

		***B***	**Wald**	***p***	**OR (95% CI)**
Gender	Male	0.333	11.744	<0.01^**^	1.395 (1.153–1.688)
School type	Junior middle school	0.723	51.327	<0.001^***^	2.060 (1.690–2.510)
Place of residence	Rural	−0.306	9.757	<0.01^***^	0.737 (0.608–0.892)
Smoking	No	−0.039	0.035	0.851	0.961 (0.637–1.450)
Alcohol drinking	No alcohol drinking	−0.517	18.284	<0.001^***^	0.596 (0.471–0.756)
Gambling	No gambling	−0.611	6.338	<0.05^*^	0.543 (0.337–0.873)
Sensation seeking		0.177	98.136	<0.001^***^	1.194 (1.153–1.237)

*The gender reference category is women; the school-type reference category is senior middle school; the native place reference category is urban; the smoking reference category is “smoking”; the alcohol drinking reference category is “drinking”; the gambling reference category is “gambling”; and the sensation seeking is a continuous variable*.

* *p < 0.05*,

** *p < 0.01, and*

*** *p < 0.001*.

## Discussion

The study investigated the relationships among extreme sports participation, sensation seeking, and negative risky behaviors (smoking, drinking alcohol, and gambling) among Chinese middle-school students. The results revealed that 19.9% of middle-school students in China have participated in extreme sports, which is higher than the proportions that have smoked (4.8%), drank alcohol (18.4%), and gambled (3.0%). Extreme sports are understood to be socially acceptable positive risky behaviors, and many kinds of extreme sports have been widely developed in many universities and primary and middle schools in China, including indoor rock climbing, skateboarding, parkour, and roller skating. Thus, the extreme sports participation rate was higher than that of smoking, drinking alcohol, and gambling (Zhao and Wang, 2009).

There were significant differences in extreme sports participation based on the gender, school type, native place, smoking, drinking alcohol, gambling, and sensation seeking (all *p* < 0.001) of students. The participation rate among boys was higher than that of girls, which was related to the fast speed and high physical consumption of many extreme sports; boys had advantages over girls in terms of their speed and physical strength; thus, they found it easier to participate in extreme sports (Su, 2015). The participation rate of junior middle-school students was higher than that of senior middle-school students, which was related to the different learning tasks of students at different stages. As senior middle-school students had to take the National College Entrance Examination, their study tasks and pressures were greater than those of their younger counterparts. As a result, they had less time and energy to participate in extreme sports. Next, compared with traditional sports, extreme sports have certain requirements in terms of venues and equipment. Most extreme sports venues are located in cities, so urban students can gain more exposure to these activities, making their participation rate higher than that of rural students. Students who engage in smoking, drinking alcohol, and gambling had higher participation rates in extreme sports than those who did not engage in these behaviors, indicating that extreme sports are associated with negative risky behaviors. Students with high levels of sensation-seeking had a higher participation rate, likely because extreme sports are dangerous, exciting, and challenging (Su, 2015), and the sports could meet the needs of students in terms of seeking these sensations.

Drinking alcohol and gambling were positively associated with participation in extreme sports. The result confirmed a part of Hypothesis 2, while smoking was not associated with participation in extreme sports. Alcohol consumption and gambling are fashionable recreational activities for adults in China (Leeman et al., 2014). However, society, schools, and families have guided adolescents to avoid drinking and gambling because these behaviors have negative effects on the physical and mental health of adolescents. Middle-school students are in a rebellious stage, and drinking and gambling can satisfy their rebellious psychology and bring them a sense of excitement (Ilk and Emt, 2014). Compared with traditional sports, extreme sports have greater risks, stimulation, and novelty, and middle-school students are willing to participate in them. The main reasons for their interest include rebellion and the pursuit of stimulation and fashion, which is consistent with the motivation of students to participate in drinking alcohol and gambling. Therefore, middle-school students who engage in drinking and gambling behaviors are likely to also participate in extreme sports.

Sensation seeking was associated with participation in extreme sports; students with a higher level of sensation seeking were more likely to engage in these activities, a result that is consistent with the previous findings (Baretta et al., 2017; Breivik et al., 2017; Frick, 2021). The result confirmed Hypothesis 1. Compared with those individuals with a low level of sensation seeking, those with a high level of sensation seeking tend to have lower risk estimations and lower anxiety levels when participating in risky and stimulating activities (Frick, 2021). Thus, high-sensation-seeking middle-school students will underestimate the risks of extreme sports and have relatively low anxiety regarding participating in these activities, which makes them more likely to participate in extreme sports compared with low-sensation-seeking middle-school students. At the same time, extreme sports can meet the sensation-seeking needs of middle-school students in several ways. First, they can gain a sense of adventure and excitement because extreme sports are more dangerous and difficult than traditional sports, and the participants need to be brave, which can meet the needs of individuals for adventure. Participating in extreme sports can arouse the positive emotional experiences of middle-school students and give them the desired stimulation (MacIntyre et al., 2019). Second, they can gain a sense of fashion and novelty. Extreme sports are novel and fashionable (such as skateboarding, roller skating, parkour, and so on), which is a popular lifestyle goal among adolescents. In the process of participating in extreme sports, middle-school students can pursue fashion and novelty (Su, 2015). Finally, through these activities, the students can meet challenges and highlight their personalities. The popularity of extreme sports has been linked to teenage rebellion. In the process of participating, the middle-school students can challenge traditional values and better show off their unique traits. Compared with traditional sports, extreme sports are more dangerous, so participating in them can make the middle-school students experience a breakthrough, highlight their personality, and gain recognition from peers (Zhang, 2013; Xue and Yan, 2018).

One limitation in this study was that the participation rate in extreme sports was investigated on the whole, but partaking in different types of extreme sports was not investigated. The results indicated that negative risky behavior and sensation-seeking were associated with practicing extreme sports, and students who drank alcohol, gambled, and sought sensations more often participated in extreme sports than their peers; these results had a reference value for extreme sports education in middle school. In future research, the participation rate, influencing factors, motivation, effects on health, and well-being of different types of extreme sports should be further explored from a psychological perspective (Brymer et al., 2020).

Based on the results of this study, we propose the following suggestions for extreme sports education in schools.First, extreme sports education should be integrated with physical education. In PE class, the teachers should lead students to participate in high safety extreme sports (such as roller skating, skateboarding, BMX, indoor rock climbing, and so on), to strengthen the extreme sports skills training, to cultivate the safety awareness of students, and to guide students to participate in extreme sports actively and safely. Second, the mental health of a teacher should carry out risky-behavior education through group psychological counseling the students for drinking and gambling. In group counseling, a teacher should guide the students to participate in extreme sports instead of drinking and gambling to satisfy their rebellious psychology and sensation-seeking needs. Third, through mental health class, PE class, and publicity board, the students are made to know that participating in extreme sports can satisfy their sensation-seeking needs. So, when they want to seek “varied, novel, complex, and intense” experiences and feelings, they will choose to participate in safe extreme sports.

## Conclusion

The participation rate of Chinese middle-school students in extreme sports is 19.9%. Students who drink alcohol and gamble and who had high levels of sensation seeking were more likely to participate in extreme sports. Middle schools should integrate extreme sports education into physical education and risky-behavior education, strengthen relevant knowledge and safety training, carry out training for extreme sports participation, improve safety awareness, and guide students to meet the needs of sensation seeking through participating in extreme sports rather than negative risky behaviors.

## Data Availability Statement

The original contributions presented in the study are included in the article/Supplementary Material, further inquiries can be directed to the corresponding author/s.

## Author Contributions

All authors listed have made a substantial, direct and intellectual contribution to the work, and approved it for publication.

## Conflict of Interest

The authors declare that the research was conducted in the absence of any commercial or financial relationships that could be construed as a potential conflict of interest.

## Publisher's Note

All claims expressed in this article are solely those of the authors and do not necessarily represent those of their affiliated organizations, or those of the publisher, the editors and the reviewers. Any product that may be evaluated in this article, or claim that may be made by its manufacturer, is not guaranteed or endorsed by the publisher.
